# The status and health burden of neurocysticercosis in Mbulu district, northern Tanzania

**DOI:** 10.1186/s13104-018-3999-9

**Published:** 2018-12-13

**Authors:** Beda John Mwang’onde, Mwita John Chacha, Gamba Nkwengulila

**Affiliations:** 1 Department of Biosciences, Solomon Mahlangu College of Science and Education, Sokoine University of Agriculture, P.O. Box 3038, Morogoro, Tanzania; 2 Department of Zoology and Wildlife Conservation, University of Dar es Salaam, P.O. Box 35064, Dar es Salaam, Tanzania; 3 Department of Zoology and Wildlife Conservation, Wellcome Trust Fellow, P.O. Box 35064, Dar es Salaam, Tanzania

**Keywords:** Human cysticercosis, Neurocysticercosis, Status, Mbulu district

## Abstract

**Objective:**

The objective of this study was to assess the extent and health burden of neurocysticercosis in the general community of the Mbulu district, northern Tanzania. About 1051 randomly select participants were screened for human cysticercosis. The Cysticercus Western Blot IgG and Computed Tomography scan were used to detect infection by cysticerci. The DALYs was used to assess the community’s health burden vis-a-vis neurocysticercosis.

**Results:**

The sero-prevalence of HCC was 16.27%. About 76% of 25 selected human cysticercosis sero-positives had neurocysticercosis suggestive lesions on CT scan and 74% had history of epilepsy. Epilepsy caused 2.8 years of life lost and 2.2 healthy years of life lost due to disability per 1000 person-years in Mbulu. The average DALYs imposed due to neurocysticercosis and epilepsy were 3.0 and 3.9 per 1000 person-years, respectively. Neurocysticercosis is a serious public health concern in northern Tanzania.

## Introduction

Neurocysticercosis (NCC) refers to infection of the brain by the larval form (cysticerci) of the pork tapeworm, *Taenia solium*. It is a form of human cysticercosis (HCC) and is the most important parasitic infection of the central nervous system (CNS) in humans [1]. NCC is unique in that the living parasite is well tolerated in the human brain [2]. Symptoms and clinical signs primarily result from death of the organism and accompanying inflammatory reactions in the human CNS [2]. The cysticerci tend to inhibit humoral and cellular immune responses as well as inflammatory reactions and cytokines, particularly IFN-γ and IL-2, and to a lesser degree IL-4 production [3, 4]. Live cysticerci also secrete cysteine and serine proteases that deplete CD4+ cells (T helper cells) that send signals to other types of immune cells, including CD8 killer cells which destroy and kill the infection [3]. The exposition of these molecules provides insights into the mechanisms by which *T. solium* cysticerci evade host immunological attack and are able to survive long periods of time [3]. Nevertheless, even if one develops neurological symptoms in early stages, it is difficult to link it with NCC. Another downside to this is that epilepsy in developing countries like Tanzania is highly associated with witchcraft, familial traits and/or a punishment by the spirits for wrong doing in the community. Ignorance on the epidemiology of NCC exacerbates public health and socioeconomic burden of the affected communities. This study highlights the status and health burden of NCC in Mbulu district for decision science.

## Main text

### Materials and methods

#### Study area

The study was carried out in Mbulu District (3.80°–4.50°S, 35.00°–36.00°E) as part of the bigger study on the status and epidemiology of *T. solium* cysticercosis in southern and northern highlands of Tanzania between March 2013 and January 2014. The district has a population of 320,279 and an average household size of 6.0 [5]. The district lies at an estimated altitude of 1000–2400 m above sea level and its climate ranges from semi-arid to sub-humid with an annual rainfall of < 400 and > 1200 mm [6]. Relative humidity ranges from 55 to 75% and mean annual temperatures range from 15 to 24 °C (*Ibid*.).

#### Study design

This was a cross-sectional study. Blood samples were collected from randomly selected and consenting individuals of different age groups and both sexes in 25 purposefully selected villages. The village executive officers in consultation with the principal investigator conducted meetings for sensitization in every village/hamlet selected for sampling. The principal investigator explained the purpose of the study. Any member of the village/hamlet aged between 0 and 80+ [7, 8] was eligible for participation. To select the study participants, each of the willing village members picked a folded piece of paper on which the words “Yes” or “No” were written. Those who picked “Yes” signed an informed consent form to participate in blood sampling. Children were sampled upon consent from their parents. Strict phlebotomy procedures were followed.

#### Sample collection and laboratory analysis

About 3 mls of cephalic venous blood was collected from each of the consenting participants using sterile plain vacutainer tubes. The samples were then centrifuged at 4800 rpm for serum extraction and the sera were aliquoted and stored in cryogenic vials at − 20 °C before use. The presence of cysticercus antibodies from sera was determined using the Cysticercus Western Blot (CYSWB) IgG (LDBIO Diagnostics 69009 Lyon—France) within 21 days of sample collection [9]. Twenty five participants were randomly selected from 48 positive participants with 5–6 well defined molecular bands on CYSWB IgG test for contrasted cerebral computed tomography (cCT scan) for NCC.

#### Computed tomography (CT) scan

The presence of cysticerci in the CNS (brain) from the cysticercosis positive participants was confirmed in a hospital by the use of single slice scanner at one tube rotation cCT scan (TOSHIBA CT Scan x-ray machine; Generator Model: TSX-003, T/No. CXXG-006, S/No. A8572114; Tube S/No. A8572162; Toshiba Medical Systems Corporation, 1998). CT scan detects ring-enhancing lesions (stage II) and multiple ring enhancing lesions (stage III) in NCC infected patients [10]. At the hospital each of the participants underwent strict medical procedures before undergoing a cCT Scan. Positive cases were referred to a neurosurgeon who prescribed medication for management of the condition.

#### Estimation of health burden

The health burden of neurocysticercosis (NCC) was estimated using the serological screening and CT scan results and the Disability Adjusted Life Years (DALYs) [7, 11, 12] in respect of the Mbulu district population [5]. The incidence was obtained from positive cases per each age group over total populations of the specific age group multiplied by 1000.

Data entry and validation was carried out using MS-Excel 2010 version (Ms Corp., Redmond, WA, USA). Descriptive analysis on HCC sero-prevalence was carried out by using Statistical Package for Social Sciences version 19.0 (SPSS Statistic 19, 2010 IBM). The unequal variance t-test (Welch’s t-test) was used to assess variations of infection between different age groups and gender, α = 0.05.

### Results

#### Prevalence and burden of human (neuro)cysticercosis

Sera were collected from 1051 individuals and the participant ratio of female to male was 1:1.7. The mean age of participants was 34.61 ± 18.4 with a range of 3–95 years. About 16.3% (171) of the sampled population in Mbulu district tested positive for HCC Abs with 75% (128) of the positive cases being males. Fifty nine percent (101) of the positive cases displayed the lower MW band (6–8 kDa) indicating an active/live cysticerci infection. The prevalence levels of human cysticercosis between age groups was not statistically different (p = 0.229). However, notable was that the ≥ 80 years age group was the least infected (p = 0.004) and the 0–4 age group had no positive case (Table 1).

**Table 1:** Seroprevalence of human cysticercosis based on age groups and sex in Mbulu District, Northern Tanzania, 2013–2014

Age group	Number tested	Total positive cases	% positive	% positive males	% positive females
0–4	5	0	0	0	0
5–14	128	14	10.9	7.03	3.9
15–29	334	44	13.2	12	1.2
30–44	284	53	18.7	14.4	6.3
45–59	168	37	22.2	15.5	6.7
60–69	75	14	18.7	8	10.7
70–79	37	8	21.6	13.5	8.1
80+	20	1	5	5	0

**Fig. 1:**
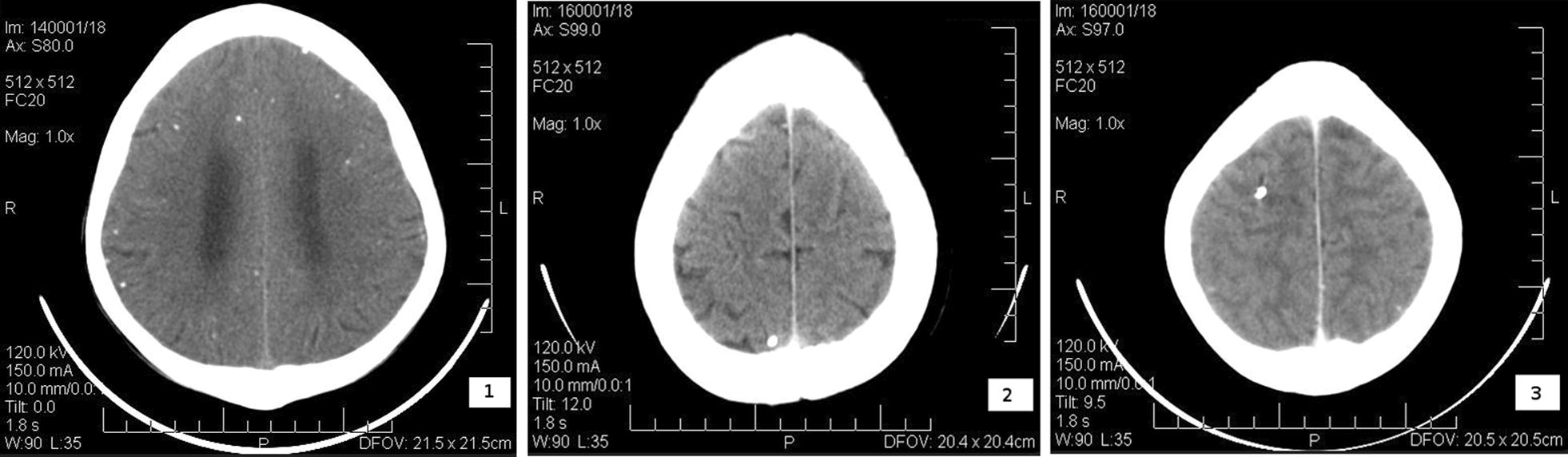
CT scans of three of the HCC sero-positive cases showing widely and diffusely spread calcifications in the brain^1^; calcification in the right occipital lobe^2^ and calcification in the left temporal-frontal lobe^3^

Of the 25 HCC sero-positives examined for NCC by CT scan, 76% (19) had one to several calcifications suggestive of NCC. Of the 19 individuals with NCC suggestive lesions, 26.3% (5) did not have any history of epilepsy but recurrent headaches. One participant among the 5 who was found with a single calcified cyst on the left frontal lobe also had multiple rings suggestive of toxoplasmosis in the brain. Seventy four percent [14] of the 19 with lesions had a history of epilepsy for a period of 2–10 years. Of the 6 with no signs of calcified cysts in the brain, 66.7% (4) had been suffering from epilepsy for 2–26 years and on further examination of one of them with frequent unprovoked seizures, it was revealed that the patient was also infected with *Brucella* spp. The remaining 2 (33.3%) were suffering from recurrent headaches for the past 2–5 years by the time they were examined (Fig. 1).

Generally, epilepsy caused 2.8 years of life lost (YLLs) and 2.2 healthy years of life lost due to disability (YLDs) per 1000 person-years in Mbulu. The YLLs was 2.8 for males as well as for females. The YLDs (0.8) for males was significantly lower than that for females (1.5) (p = 0.001). The DALYs due to epilepsy was 3.9 per 1000 person-years, representing the disease burden in Mbulu district. The DALYs imposed by NCC on rural communities in Mbulu was estimated to be 3.0 per 1000 person-years.

### Discussion

The findings of this study show that the prevalence and the burden caused by NCC related epilepsy is considerably high in Mbulu district. The proportion of those exposed to infection by *T. solium* cysticerci in humans, based on circulating anticysticercal antibodies (Abs) is as high as one person in every six members of the community in the district. Furthermore, cCT scan results from selected sero-positive individuals showed that a significant proportion of them had NCC suggestive lesions with over 56% of them being epileptic. Even though the results of this study cannot be generalized, the number of sero-positive individuals with confirmed NCC suggestive lesions is a strong pointer to the contribution of the disease to epilepsy syndrome in the district. This prevalence level in the northern part of Tanzania is similar to that reported in the southern highlands of the country [13]. On the other hand, the observed prevalence in the present study was higher than that reported in Rwanda (2.8%) [14] and in Mozambique (12.1%) [15]. Furthermore, the observed prevalence is significantly less than that reported in Burundi (31.5%) [16]. While all HCC results compared in here are from the same region i.e. Africa south of the Sahara, the observed differences might be attributed to differences in the target populations studied, methods used for diagnosis/surveillance and the prevalent risk factors in each particular area.

In the present study, the prevalence of HCC was significantly higher in males than females. In Mbulu district the population of males in 2012 was higher than females [5]. The participant ratio for females vis-a-vis males was 1:1.7. Besides, Mbulu district is dominated by agro-pastoral communities. Under such a mode of life, communities’ roles between males and females are distinctly well known [17]. Yet, in such communities, women before menopause hardly participate in social functions and therefore might not suffer the same exposure levels like their husbands. The prevalence of NCC lesions established by this study was alarming! The high prevalence of NCC might be contributed by high preference of *T. solium* cysticerci in CNS [1].

The transmission of HCC/NCC might be attributed to the life style of the Mbulu community [13]. Some households do not share toilets with their daughters and mothers in-laws thus heads of households prefer defecating out in the bush. In this area, pigs are kept under free-range systems thus facilitating the cycle of this disease.

The burden caused by NCC related epilepsy was considerably high in Mbulu district when compared to the WHO estimates, 29% [18]. The differences in proportions of NCC associated epilepsy might be due to research approaches in establishing the prevalence. For example studies by Bhattarai et al. [12], Praet et al. [19] and Carabin et al. [20], where WHO rely under this case, were based on systematic reviews and meta-analysis data. Such studies are very important in making policy decisions although they are limited by selection of studies, limitations in executions of design and methods used to obtain primary data and heterogeneity. Bhattarai et al. also used stratified data from urban areas where the risk of acquiring *T. solium* eggs is relatively lower compared to rural areas where sanitation and hygiene standards may be questionable. This could be the reason that the proportion of NCC associated epilepsy in developing countries was estimated at 29%. The current study was cross-sectional. Data were obtained from the general community and that might be reporting the situation in rural poor because the scant studies related to NCC/HCC in Tanzania are hospital based or are focused to people with epilepsy.

As CNS syndromes of unknown origin is kept on increasing in cysticercosis endemic regions, wider and broad studies from different countries and regions in order to control *T. solium* cysticercosis are of paramount importance. It must also be noted that diagnosis challenges for HCC might be a limiting factor towards achieving this goal.

## Limitations of the study

The results of this study may not be generalized to a wider population because it focused on agro-pastoral communities limited to a district. However, the study mainly depended on exposure seroprevalence and suggestive lesions diagnosed via CT Scan as confirmatory test.
